# GIS-based analysis of field margin photovoltaic potential at the landscape level in northwestern Germany

**DOI:** 10.1038/s41598-026-48425-2

**Published:** 2026-04-24

**Authors:** Hannes Foth, Roland Pesch, Broder Breckling

**Affiliations:** 1 Institute for Applied Photogrammetry and Geoinformatics, Jade University of Applied Sciences, Ofener Str. 16/19, 26121 Oldenburg, Germany; 2 Faculty II, University of Vechta, Driverstraße 22, 49377 Vechta, Germany

**Keywords:** Field margin, Photovoltaics, GIS, Automated geoprocessing, Workflow model, Ground truthing, Ecology, Environmental sciences, Energy science and technology

## Abstract

Climate change and other factors require transformations of the agricultural sector towards sustainable land use practice. The integration of renewable energy systems, particularly photovoltaics, into agricultural and rural land-use systems receives increased attention to decarbonise resource use and consumption and to stabilise incomes and livelihoods in rural regions. This study analyses the quantitative potential of a new concept, *Field Margin Photovoltaics*, in north-western Lower Saxony, Germany. This concept combines crop cultivation, photovoltaics and biodiversity promotion with wildflower strips and/or hedges. We use a GIS workflow to quantify marginal structures from landscape models, relying on available geospatial data from regional authorities. The marginal structures are categorised based on suitability criteria and solar exposure to estimate the potential energy yield in the region. The results of a regional analysis show that field margins allow a significant contribution to renewable energy production without significantly compromising other land uses. For a region of almost 15,000 square kilometres, more than 1.3 million field margin sections with solar potential were considered. The total length of potentially suitable margins was calculated to be 97,870 km. About 27,300 km were predicted to be partly shaded. Estimating the integration of photovoltaic modules with a surface area of one to four square metres per metre of field margin would result in a theoretical maximum energy potential of around 119 TWh. The modelling work is part of a transdisciplinary project to address various aspects of transformation research (www.vier-n.de). The GIS analyses presented here serve for regional planning and stakeholder discourses together with a living lab approach.

**Supplementary Information:**

The online version contains supplementary material available at 10.1038/s41598-026-48425-2.

## Introduction

Measures to mitigate the impacts of climate change are a cross-sectoral task for society as a whole that requires transformations towards a higher degree of sustainability in order to ensure the maintenance and further development of social, economic and ecological well-being^1^. Efforts to decarbonise the energy supply, reduce fossil fuel use and replace it with renewable energy systems^2^ are a particularly high priority with photovoltaics playing an important role. In an interdisciplinary project, we are evaluating new, previously unexplored opportunities and potentials for integrating photovoltaics into agricultural landscapes^3^.

In addition to systems on the roofs of agricultural buildings, two types of photovoltaic systems are common in rural regions to date: On the one hand, there are ground-mounted systems where the area is completely taken up by photovoltaic modules, making it unavailable for other uses. This leads to competition for land with agricultural uses^4^. This competition is currently one of the limiting factors for the expansion of energy generation through photovoltaics and implies vehement conflicts of interest in rural areas between the energy industry and food production. On the other hand, there are types of systems that are summarised under the term agrophotovoltaics. Agrophotovoltaics systems are categorised into two main types: ground-mounted and high-mounted. In ground-mounted systems, the panels are spaced further apart to allow agricultural vehicles to drive between the rows of modules^5^. This results in a slightly higher yield loss than with high-mounted systems. In these systems, the panels are mounted at a sufficiently high level that shade-tolerant crops can be grown underneath, thus allowing a dual use of the area. The problem with this is that the construction costs are considerable, so that they are more likely to be considered on smaller fields and for special crops^6^. Overall capacities for different installation types of photovoltaic systems under different scenario conditions have been estimated by^7,8^.

In our work, we are investigating a photovoltaic configuration that has not yet been considered: field margin photovoltaics. The panels are not installed over the entire area, but only along the least agriculturally productive borders. This only marginally restricts the established agricultural use, but at the same time makes it possible to use the strip under the panels to establish elements of wild flora, as was previously only realised as a temporary and partial set-aside of agricultural land in the EU under the flowering strip support programme^9^. The establishment of field margin photovoltaics would create permanent corridors for the spread of wild species, which would promote biotope connectivity more efficiently than short-term flowering strips of the established agroecological programmes. Sensitive border structures, e.g. along the edges of watercourses, would also be better protected. At the same time, longer-term, calculable, income-generating structures are created, which provide a significant impetus to expand technically innovative aspects of land use based on increased energy autonomy for farms.

So far, such a concept of combined land use from traditional established crops, the generation of a stable, calculable additional income even for smaller farms and at the same time the promotion of biodiversity, which is in sharp decline in the agricultural area, is new and innovative, so in this context questions of integration into the landscape framework require an in-depth analysis. The model developments documented here provide an initial information base. The model can be transferred in the same form to other regions.

We are taking steps here to explore the potential of this innovative concept and to assess how different, previously competing sustainability goals can be combined at different levels, representing both, a protective function for the productive function and a stabilising element in the landscape. As part of the development of the model, we are pursuing the following objective: to quantify possible border structures from landscape models, to apply suitability criteria from the landscape context and thus to estimate the potential for regenerative energy. This will open up a further aspect in the discussion on alternative concepts for energy sustainability in agricultural production.

We want to initiate an exchange on both the achievable benefits and the limiting factors. As part of the project, this will take place both at the conceptual, and regional modelling level and in a real-world laboratory approach that will include additional elements of practical testing alongside the modelling work.

## Materials and methods

### Study area

**Fig. 1:**
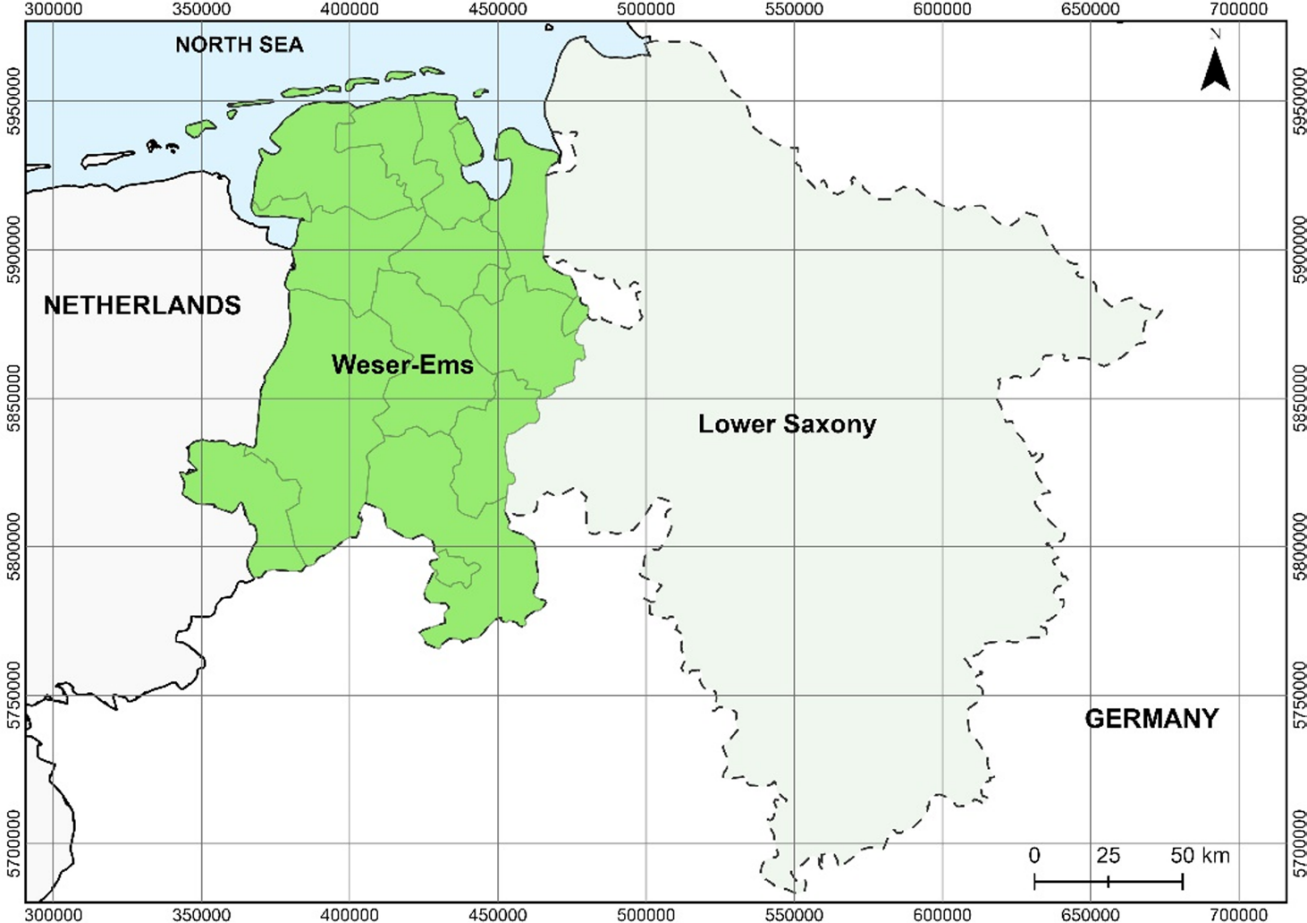
Study area is the Weser-Ems region in the western part of Lower Saxony. Data source:^10^.

The assessment for photovoltaic installations on marginal strips was conducted for the Weser-Ems region in northwestern Lower Saxony, Germany (Fig. 1).

The region extends over a width of about 110 km and a length of almost 200 km ranging from the North Sea to the northern parts of the low mountain ranges in the south. Next to the Wadden Sea with its Eastern Frisian islands, the large distribution of peat lands, especially raised bogs, is another characteristic of the region^11^.

**Fig. 2:**
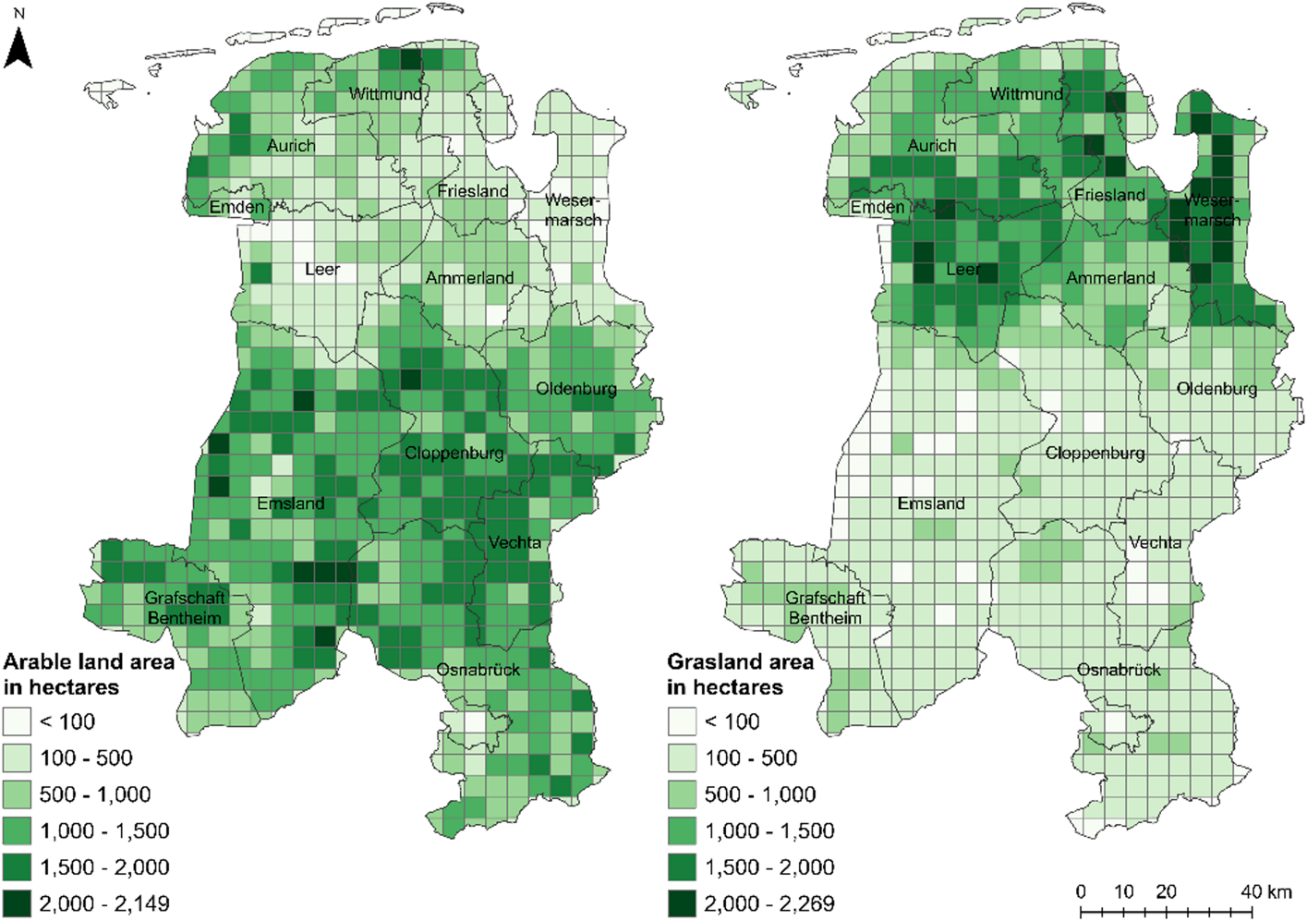
Agricultural land use patterns in the Weser-Ems region. Data source:^10,12^

The area of the Weser-Ems region encompasses 41% arable land (609,000 hectares) and 27% grassland (401,000 hectares)^12^. Figure 2 presents an aggregation of both arable (illustrated on the left) and grassland (illustrated on the right) areas for a raster with a spatial resolution of 5 km: A clear regional division observed above the districts of Emsland, Cloppenburg, and Oldenburg. Both arable land and grassland play a dominant role in the Weser-Ems region. Hence, their use for the construction of photovoltaic systems is limited. Nevertheless, global solar radiation in Lower Saxony has seen an increase over recent years. Between 1983 and 1990, the average annual global solar radiation amounted to 953.1 kWh/m2, which increased to 1,052.5 kWh/m2 between 2011 and 2020^13^. The application of field margin photovoltaics could therefore present potential opportunities for the expansion of renewable energies in the northern part of Germany.

### Data and methods

**Fig. 3:**
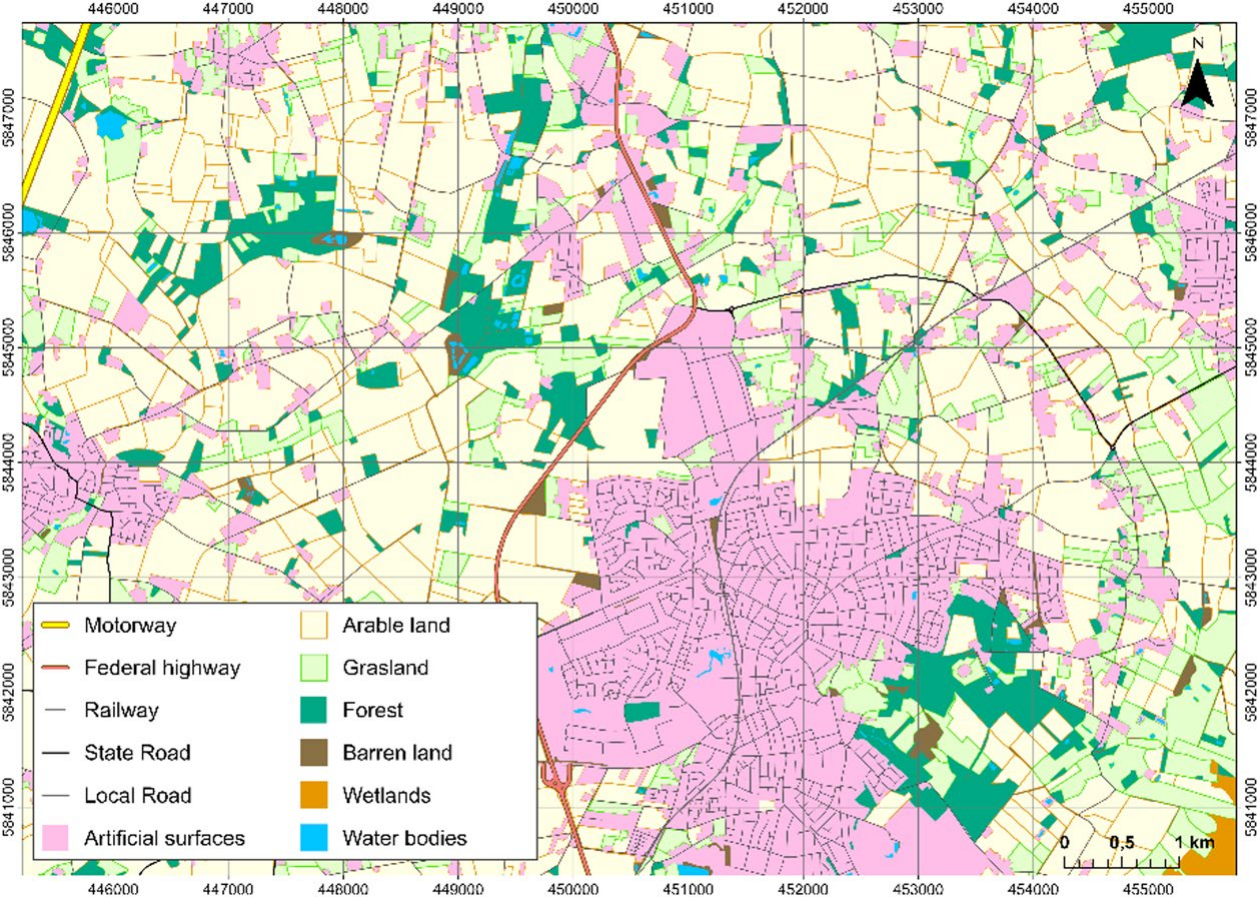
A selection of Basic-DLM objects of the Vechta region based on the 2022 dataset. This dataset was used for the entire region under consideration. Data source:^12^

Next to administrative boundaries on the municipality level^10^ the geodata included in the analysis consisted of the vector-based Digital Basic-Landscape Model (Basic-DLM) (Fig. 3) provided by the State Office for Geoinformation and Land Surveying Lower Saxony (Landesamt für Geoinformation und Landesvermessung Niedersachsen, LGLN)^12^. As a product of the Authorative Topographic-Cartographic Information System (ATKIS), the Basic-DLM is derived from topographical maps and updated by digital orthophotos (DOP) describing topographic objects as vector geometries via a standardised object catalogue with over 130 object types and a map scale of approximately 1:25,000. The vector data are projected in the ETRS89/UTM 32 N reference system and are characterised by high geometric accuracy, with linear objects having a positional accuracy of ± 3 m. The landscape model is updated every three years and allows for the identification of all arable land and grassland or forest areas. Hence, it is well suited for a detailed analysis of landscape characteristics in relation to field margin photovoltaics.

DOPs provided by the LGLN, covering the entire area of Lower Saxony, were used as a further ATKIS data product^14^. These images are equalised and true-to-scale photographic representations of the earth’s surface, with a spatial resolution of 20 cm. Georeferencing is also conducted in ETRS89/UTM 32 N. In this study, DOPs were used as a background layer to validate vector-based representations of the earth’s surface and to ground truth locations for field margin photovoltaics in GIS. The LGLN Open Geodata Portal provides free access to all data used in this study.

Different orientations (deviation from the south orientation) and tilt angles of the photovoltaic system affect the solar energy yield of photovoltaic modules. To quantify the global irradiance a photovoltaic yield table produced by^15^ was applied in our analyses. From this table photovoltaic yields can be obtained according to different orientations and tilts.

To investigate the potential of agricultural field margins for photovoltaics an extensive geoprocessing workflow was implemented in a Geographical Information System (GIS). ArcGIS Pro 3.2 (Redland, CA) was used. The ArcGIS Model Builder enables to connect sequences of processes and geoprocessing tools, allowing reruns with updated or new data models for repetitive GIS tasks. The models produced further ensure the documentation of the geodata analysis performed enabling reproducibility of the modelling results and the workflow transfer and translation to other applications, data, and regions.

The model developed to identify margins for photovoltaics considers several parameters including the selection of the study area, the type of vegetation, and the size of shaded areas, which are caused by wooded areas and tree rows along margins, which exclude their use for photovoltaics. Shaded sections were excluded through geometry-based analysis. No legal or land-use restrictions were applied, as the aim was to assess the theoretical maximum potential under ideal technical and topographical conditions. The considered width of the field margins is 5 m according to the established regulations for wildflower strips^16^.

Furthermore, a value range was defined that specifies the necessary orientation in degrees in which field margins located to the south of forests and tree rows must be situated so that they still receive sufficient sunlight for a sufficient yield. The field margins as determined by the model were subsequently categorised using the Basic-DLM objects. In addition to the exact location and length, the surrounding land use, such as nearby water bodies, settlement areas, motorways, or whether the field margin is located in a protected zone like a nature reserve, was obtained from the database for all field margins.

Based on these characteristics, the lengths of field margins were quantified for various surrounding land use categories, including those along highways and railways, as these areas are particularly suitable for the installation of photovoltaic systems under current legislation. Field margins located within 500 m of motorways and railway lines were selected using a buffer generated around the respective ATKIS infrastructure segments. All field margin segments intersecting this buffer were subsequently aggregated within a raster grid with a cell size of 5 × 5 km².

## Results

### Automated geoprocessing workflow

The workflow implemented in the ArcGIS Model Builder consists of overall 40 processing steps which are structured into three main parts:

1. Linear field margin extraction for marginal arable and grassland strips from the Basic-DLM.
2. Attribution of shading impacts from wooded areas to the field margins.
3. Quantification of solar energy potential for the field margins.

**Fig. 4:**
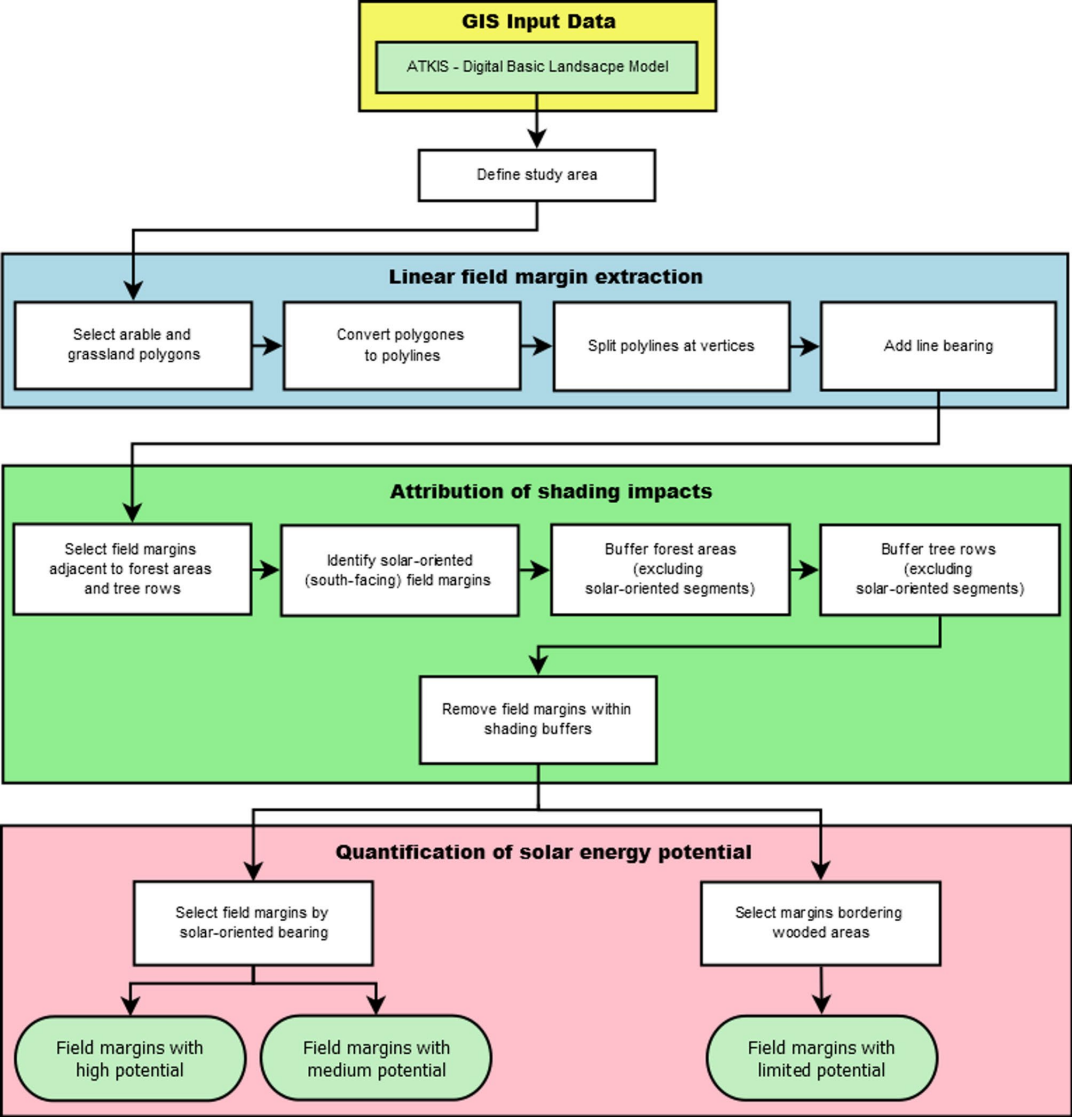
Workflow for identifying field margins with solar potential in the Weser-Ems region, modelled in ArcGIS Model Builder using Basic-DLM data.

Figure 4 provides a schematic overview of the implemented workflow, shown as a flowchart. The Supplementary Data includes the relevant ArcGIS toolbox as well as a standalone Python script version of the workflow^17^.

**Fig. 5:**
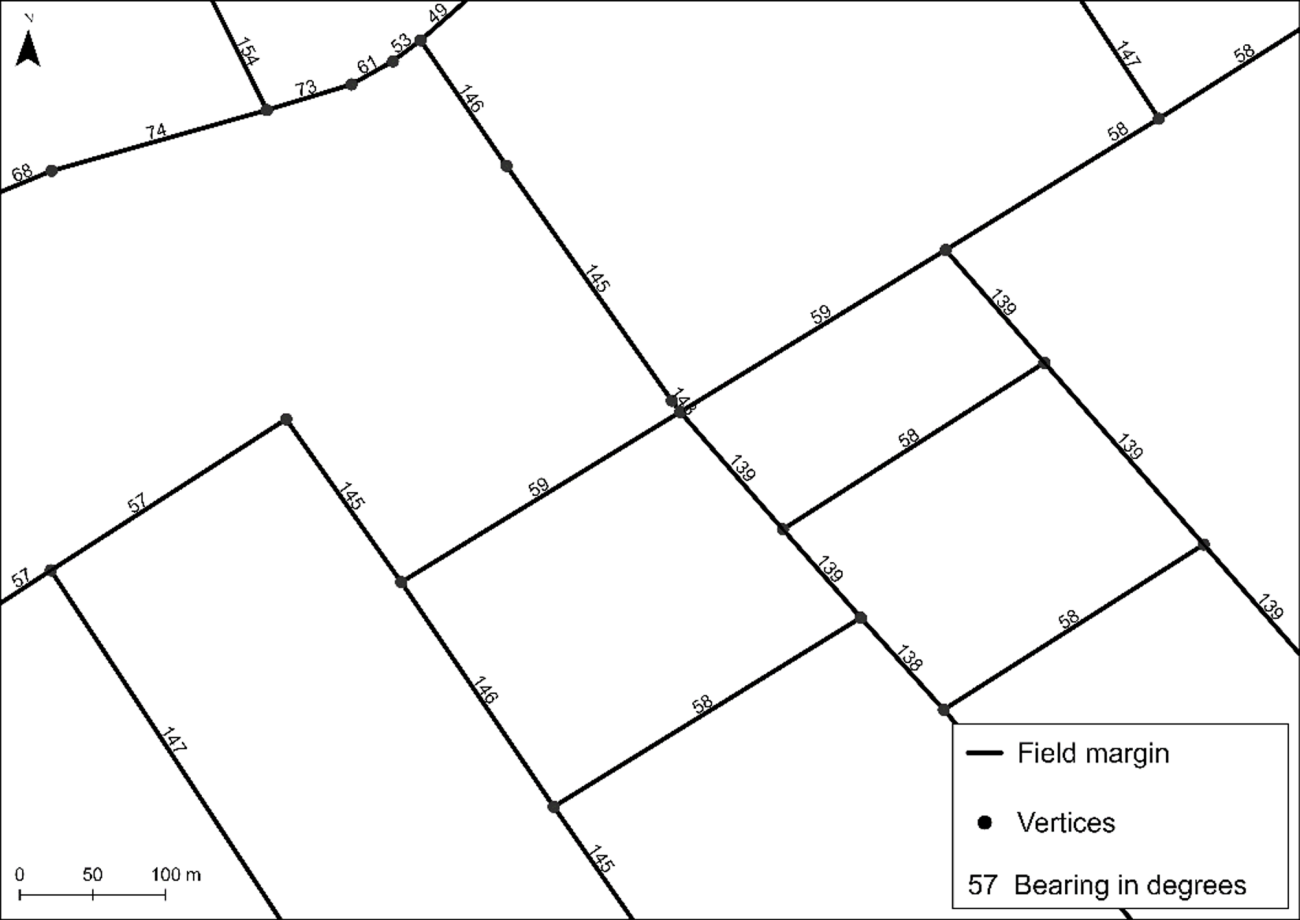
Field margins extracted from the Basic-DLM. The figure shows the bearing of each straight line representing the field margins. Data source:^12^

**Linear field margin extraction.** For the extraction of the linear field margins, all relevant polygon objects are at first selected via attribute query for arable land and grassland from the attribute data of the Basic-DLM. The resulting polygons are then converted to polyline objects which are split at their vertices resulting in the field margin strips for each agricultural field. To enable a quantification of the solar energy potential, each line object is attributed regarding its bearing using the GIS tool *Add Geometry Attributes.* This tool can assign new geometric properties to features (Fig. 5). The bearing indicates the direction of a line measured clockwise from the north. North is given as 0 degrees.

**Shading impacts.** The next processing steps are to identify all line segments that border forest areas and still have sufficient solar potential for photovoltaic installations.

All line segments bordering forests are first selected. From these, all line segments are extracted lying in the southern parts of the forest areas with bearings between 55° and 128°. The selected bearing range ensures at least ten hours of direct sunlight on representative summer days (30 April and 13 August). For shadow simulations, calculations with these days provide a more meaningful value than, for example, the longest day of the year, 21 June^18^.

The corresponding bearing range is determined from sunrise and sunset times and the corresponding solar azimuth angles of the average summer days for the centroid of the study area. The centroid is calculated as the average of all x and y coordinates of the land area, which can be implemented using the *Feature to Point* tool included in the ArcGIS software. As an example, the centroid of the Weser-Ems region is at 52°56’23’’N, 7° 46’1’’E, about 10 km southeast of Friesoythe.

**Fig. 6:**
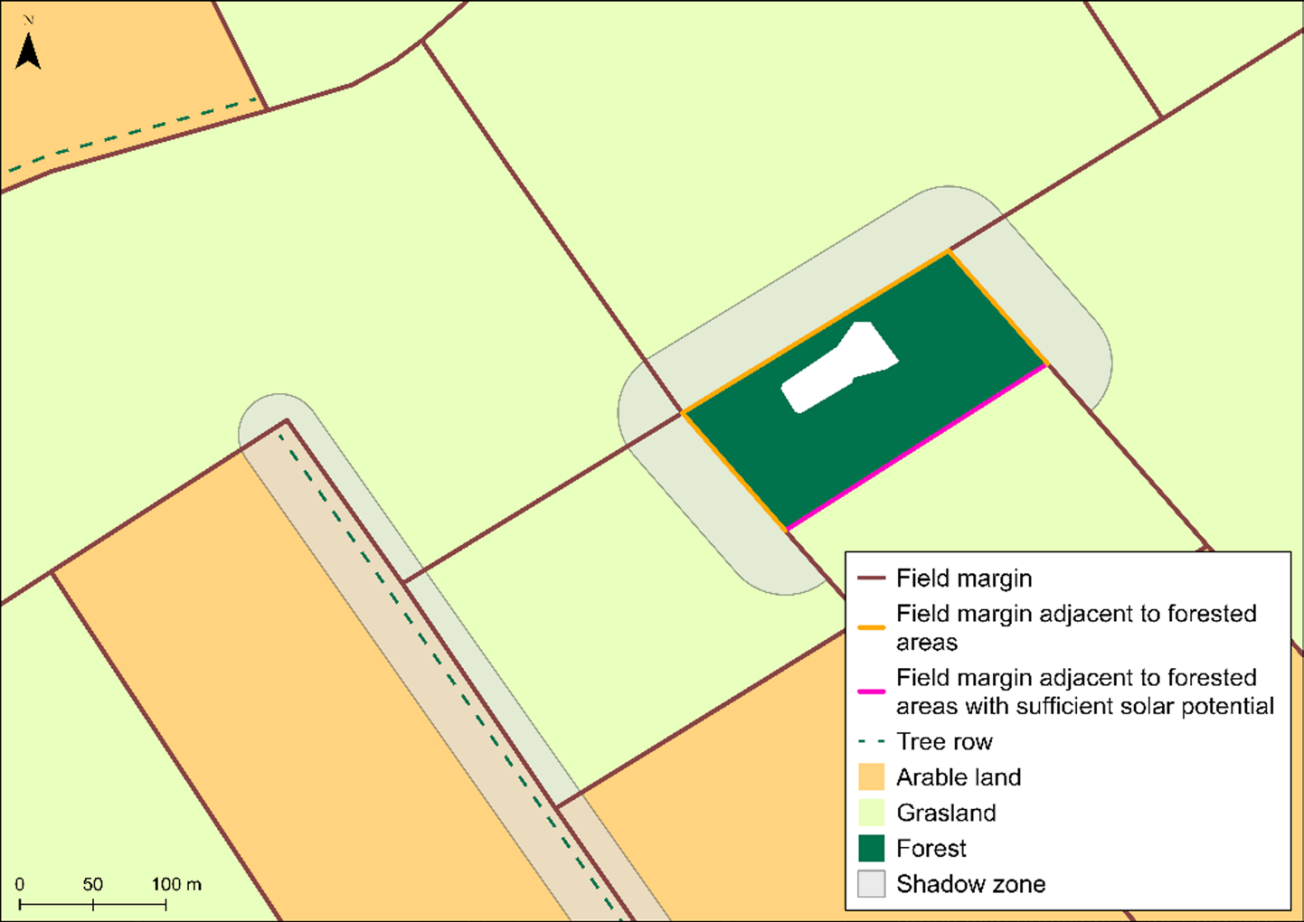
Accounting for shading effects in the selection of suitable field margins for photovoltaic. Data source:^12^

As the Basic-DLM also contains tree rows as geospatial objects, these are included in the analysis to account for shading effects to be included in the assessment of the photovoltaic efficiency. Shading is achieved by systematically excluding field margin segments affected by tree cover and forest edges. Buffers are created around forest areas and tree rows, except for south-facing forest edges and tree rows bearing between 55° and 128°. These buffer distances were determined using shadow length calculations based on typical tree heights and the average solar elevation angle on a representative summer day (13 August) in the Weser-Ems region. An average height of 15 m was assumed for tree rows and 25 m for forest areas. The solar elevation angle was calculated as the mean of the hourly solar altitude values between sunrise and sunset at the geographical centre of the region, giving an average of 29.7°. Shadow length was calculated by dividing object height by the tangent of the solar elevation angle (L = H / tan α), where H denotes object height and α denotes solar altitude. This resulted in shadow lengths of 28 m for tree rows and 44 m for forest areas, which were applied as buffer distances. Field margins affected by shading are excluded from the analysis (see Fig. 6). The buffer parameters are configurable within the model and can be adjusted for other regions according to local vegetation characteristics. No additional restrictions, such as legal or land-use constraints, were applied in the analysis.

**Fig. 7:**
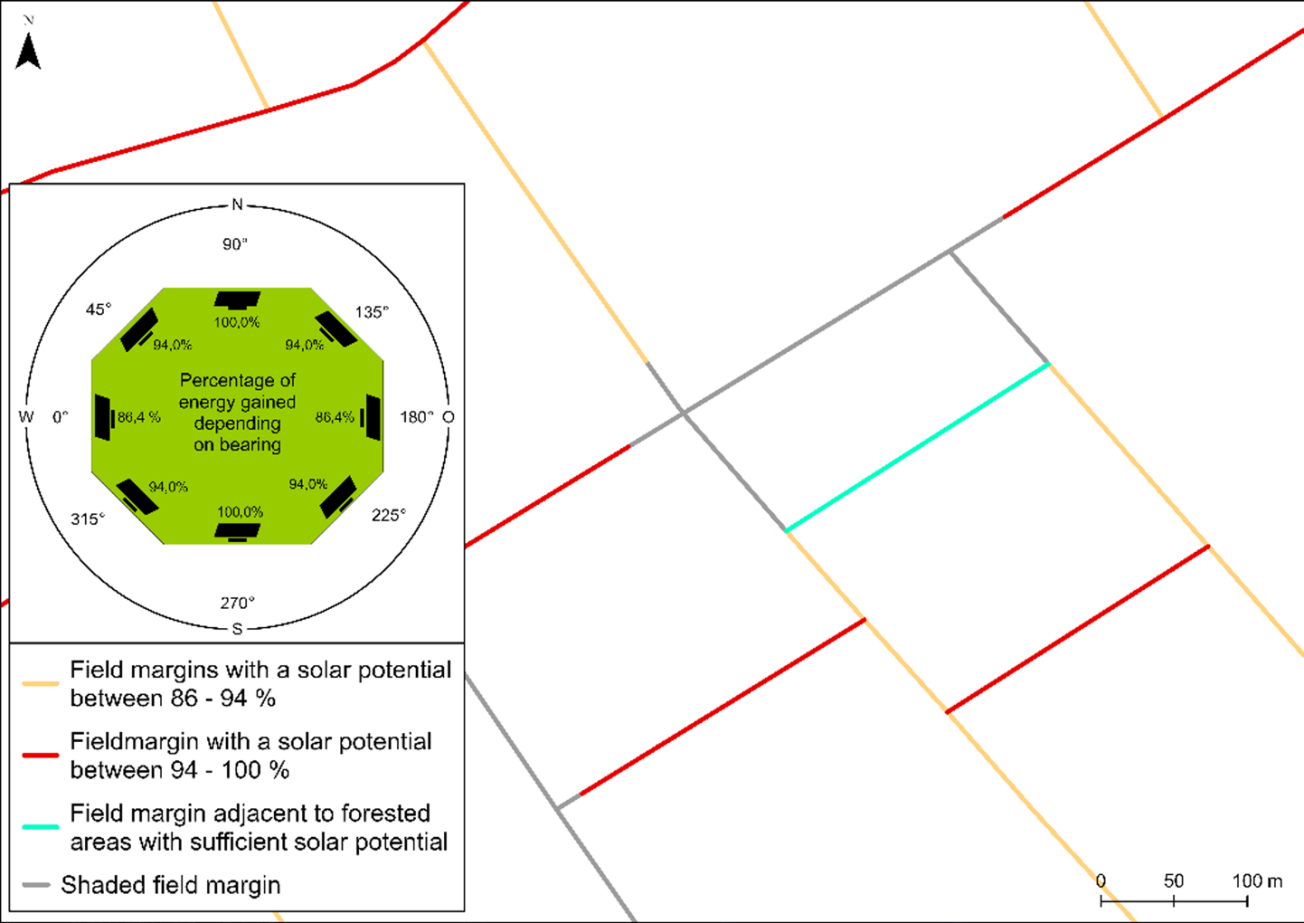
Solar classification based on bearing and adjacent woodland. A bearing of 90 or 270 degrees results in a solar potential of 100% for a field margin due to the exact southern orientation of the photovoltaic modules. A deviation of up to 45 degrees reduces the solar potential to 94% (red lines). A deviation of up to 90 degrees reduces the solar potential to 86.4% (yellow lines). Turquoise lines represent field margins that are oriented to receive sufficient sunlight despite being adjacent to wooded areas. The grey lines are field margins that are shaded by forests and rows of trees. Data source:^12^

**Quantification of solar potential.** The final step in the process is to classify the field margins based on their solar energy potential (Fig. 7). A photovoltaic system achieves the highest energy yield when oriented directly southward. In this context, field margins with an east-west alignment, corresponding to bearings of 90 degrees or 270 degrees, which represent the same orientation axis in opposite directions, offer the most favourable orientation for south-facing photovoltaic installations. Any deviation from this orientation tends to result in a decreased energy yield. The energy yield percentages were determined using the photovoltaic yield table by^15^, assuming that the panels are at their optimum tilt. Accordingly, three classes of solar potential are defined:

1. Field margins with a solar potential between 94 and 100%.
2. Field margins with a solar potential between 86 and 94%.
3. Field margins adjacent to forested areas, acknowledging the impact of shading.

### Case study Weser-Ems region (north-west lower saxony)

**Fig. 8:**
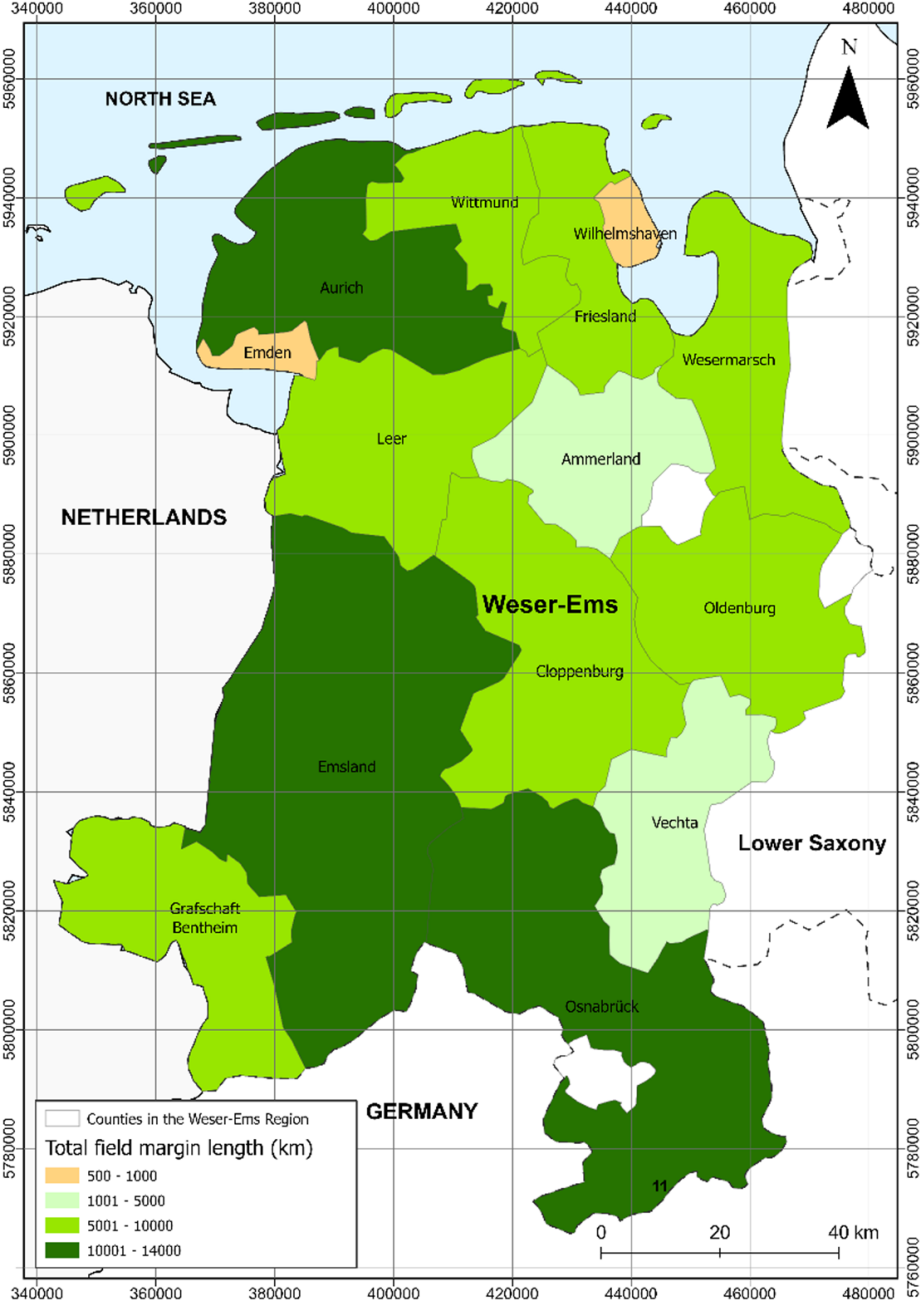
Spatial distribution of the total field margin length (km) per county in the Weser-Ems Region. Data source:^10,12^

The developed GIS workflow was applied to the digital landscape data of the Weser-Ems Region resulting in more than 1.3 million field margin sections with solar potential. The total length of suitable field margins added up to 97,870 km. About 27,300 km were predicted to be shaded. This would result in a theoretical maximum energy potential of 119.2 TWh. The calculation is based on the current assumption of an installed photovoltaic capacity of 1.35 kWp per m of field margin^19^. This corresponds to configuration that allows for the viable integration of 4 m^2^ of photovoltaic modules per meter of field margin length. However, this value represents a technical maximum and may change with future efficiency gains or site-specific installation parameters, particularly the collector surface area installed per meter. The spatial distribution of total field margin lengths across the Weser-Ems region is shown in Fig. 8. With a total of 13,846 km, the district of Emsland has the largest potential field margin length for photovoltaics, followed by the districts of Osnabrück (12,829 km) and Aurich (11,596 km). Field margins with 94 to 100% solar potential (46,847 km) and field margins with 86 to 94% solar potential (47,747 km) are roughly balanced. There are 3,276 km of potential field margins south of forest areas.

**Fig. 9:**
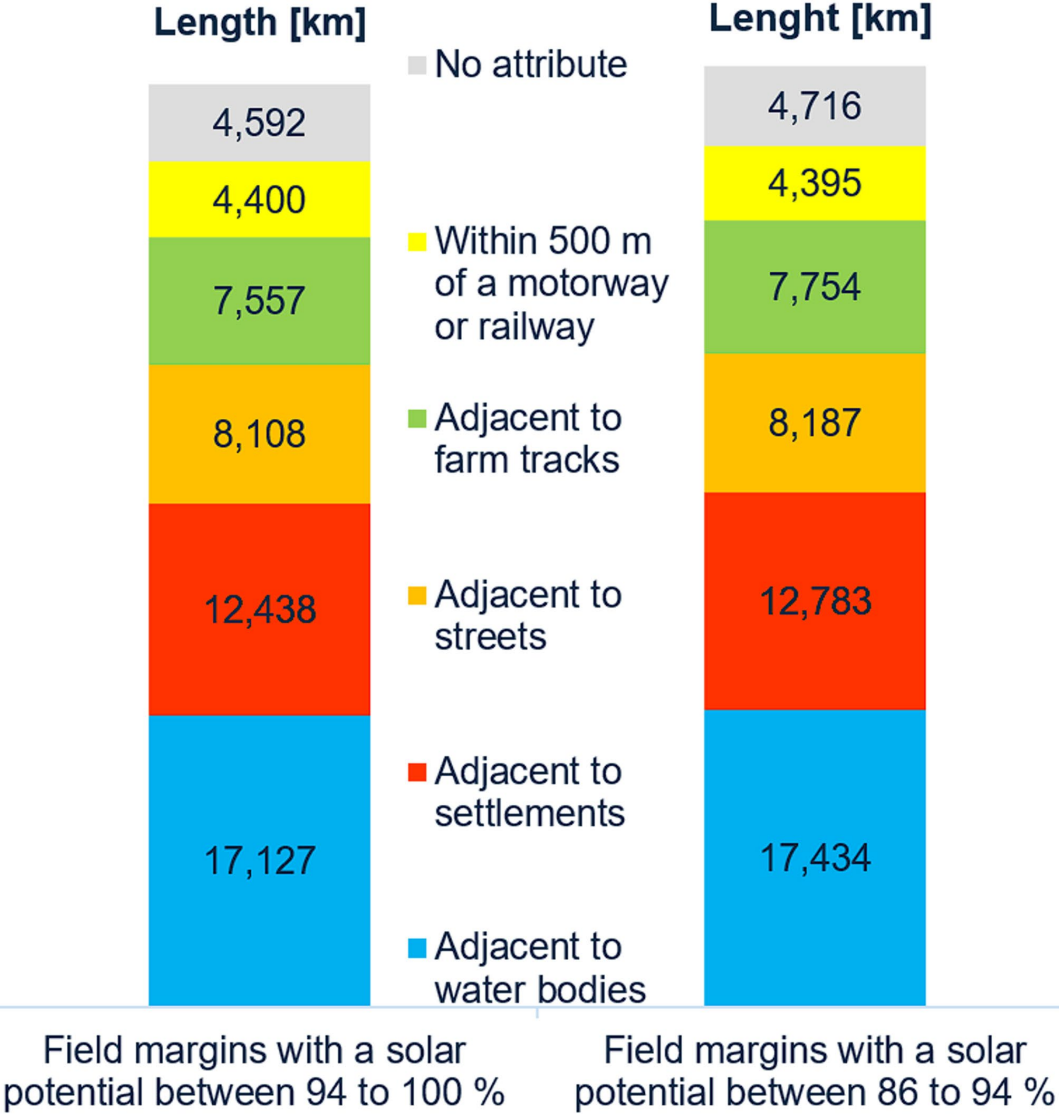
Field margin length by attribute type.

In addition to the solar potential class, further attributes were added to the field margins based on the Basic-DLM. These attributes can be used to identify suitable locations according to various criteria. Figure 9 shows the distribution of field margin lengths by attribute type.

**Fig. 10:**
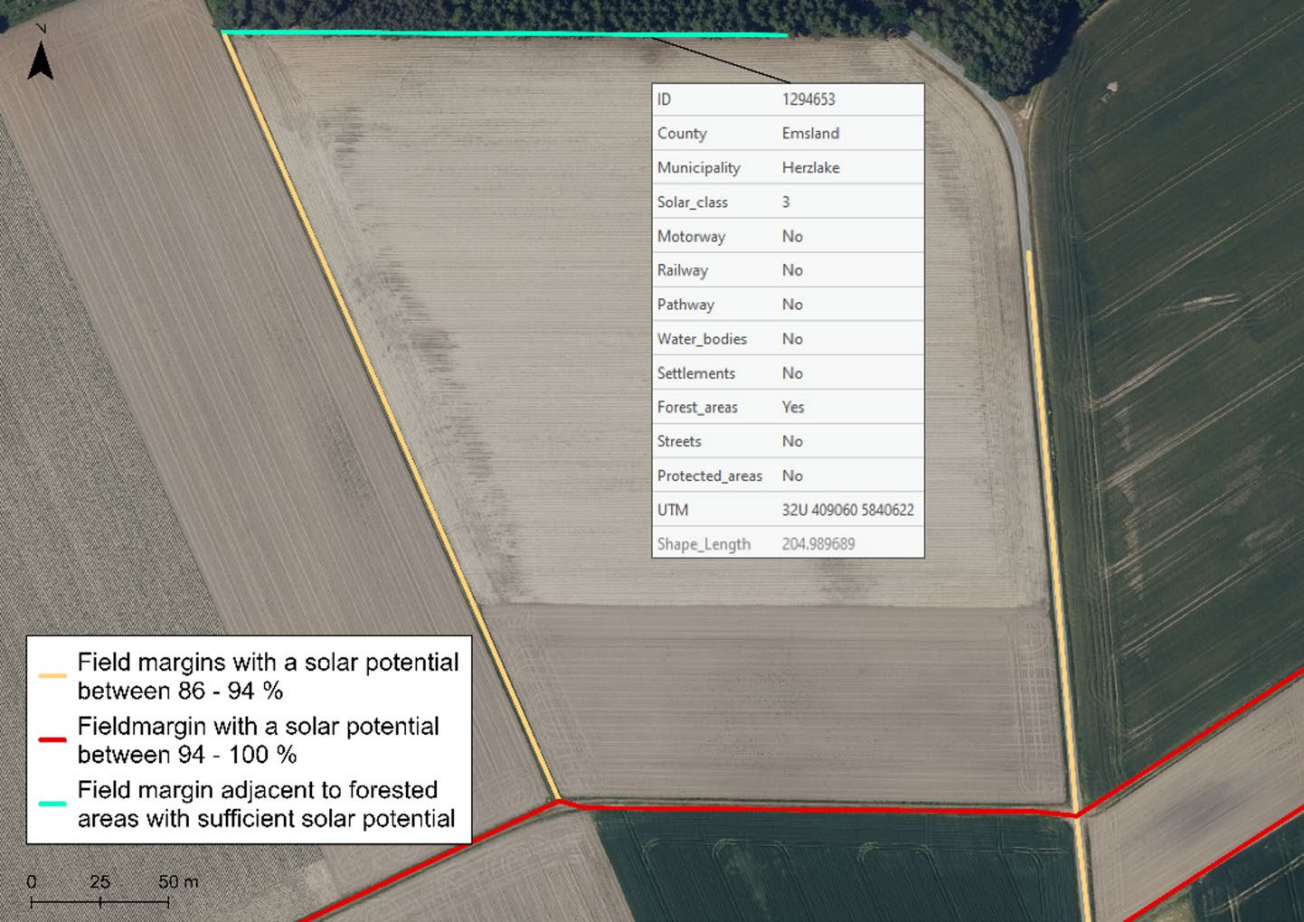
Digital orthophoto underlaid on field margin feature-class in GIS. Data source:^12,14^

There are no significant differences between the attribute types of the two classes. Field margins selected by specific criteria can be displayed in GIS map view and overlaid on DOPs for a closer look at the surroundings (cf. Fig. 10).

**Fig. 11:**
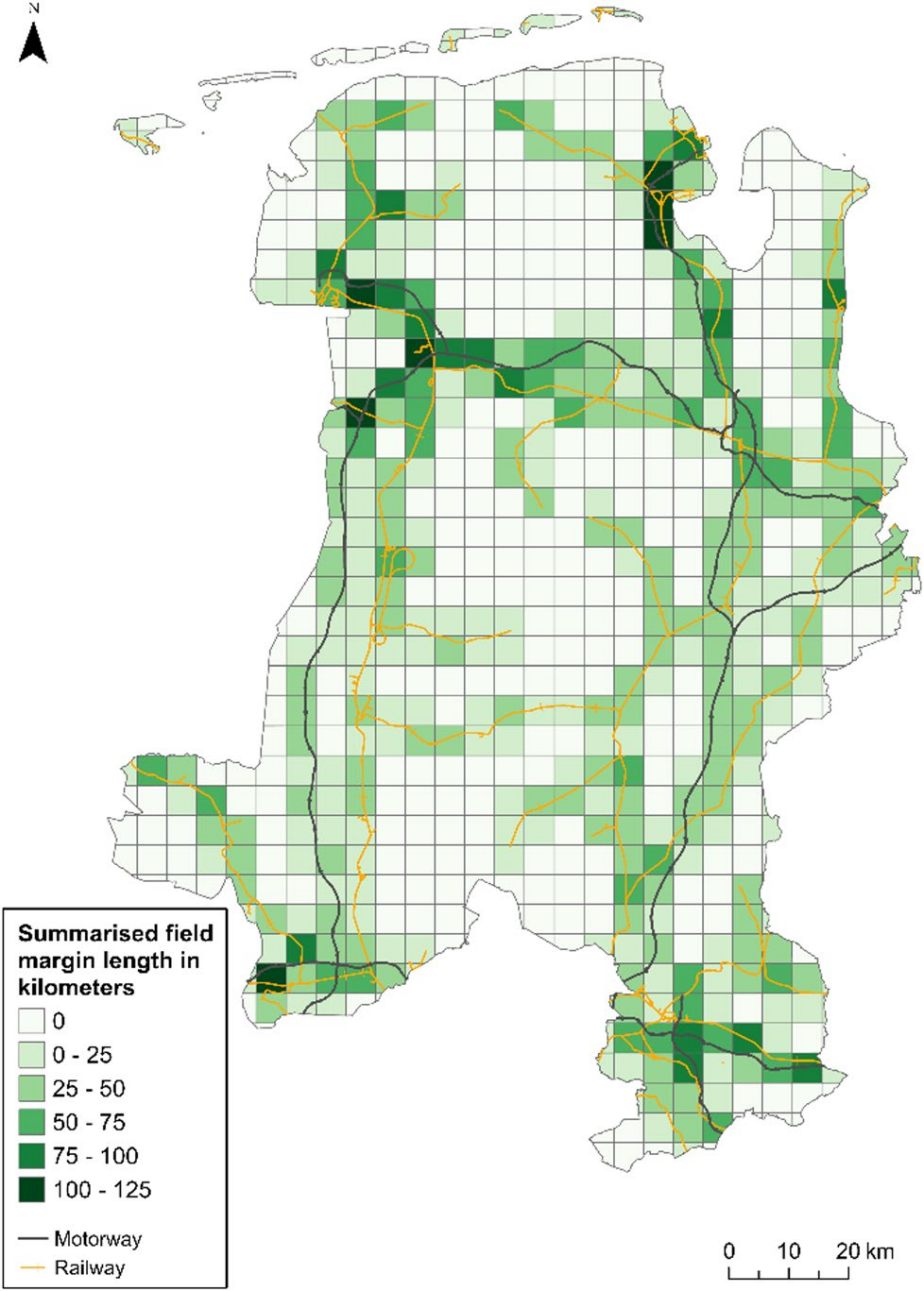
Map of aggregated field margin length within 500 m of a motorway or railway line. Data source^10,12^.

In Lower Saxony, the expansion of ground-mounted photovoltaic systems is planned in 500-metre-wide strips along motorways and railroad lines^20^. The even distribution of the motorway and rail network throughout the Weser-Ems region provides a potential opportunity for the use of field margin photovoltaics in all areas. Figure 11 shows an aggregation of the length of the field margins within 500 m of a motorway or railway line for a raster with a grid cell size of 5 * 5 km.

### Ground truthing

**Fig. 12:**
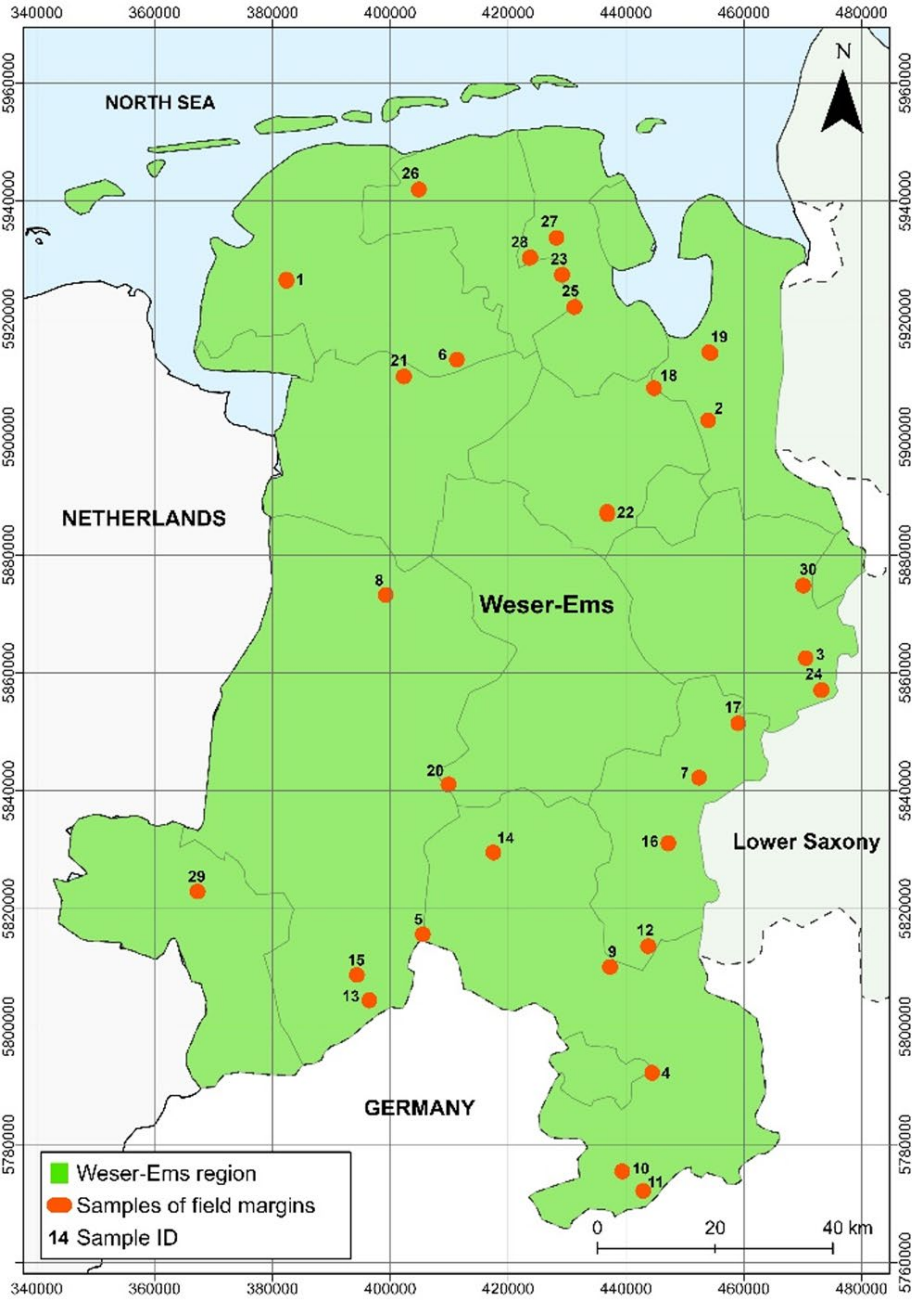
Distribution of the 30 sampled field margins. Data source:^10,12^

To evaluate the corresponding outputs, extensive ground truthing was carried out using of high resolution and recent DOPs made available by the LGLN (Fig. 10). For the ground truthing process, a series of criteria was set up and then applied to an extraction of the field margins of the Weser-Ems Region. Thereby, it was aimed to evaluate a representative sample from the area of interest consisting of 30 ground truth observations. Figure 12 shows the distribution of the samples in the Weser-Ems region. Representativity was accounted for by stratifying the entire sample to adjacent pathways, streets, water bodies, settlements, and forest areas, as well as the proximity of motorways and railways. Based on these criteria, the field margins were subjected to multivariate cluster analysis in the GIS using the *k-means* method. From the results of the cluster analysis, 30 random samples were selected using an R script. The *sample function* was used to randomly select the samples from each cluster. The number of selected field margins per cluster was determined according to the percentage of each cluster in the total size of all clusters.

Ground truthing showed that the attributes from the model matched the DOPs verification by 97% (Table 1). This suggests that the added field margin attributes from the model were reliable and provided a good representation of the actual terrain conditions. In addition, the ground truthing process estimated the percentage of shading from adjacent objects. The average shading of the sample was 20.5%. This is due to neighbouring buildings or trees not captured in the Basic-DLM. Overall, these results support confidence in the accuracy and applicability of the model for analysis and planning in the context of field margin photovoltaics.

## Discussion

To proceed with field margin photovoltaics as future components in a sustainable agricultural landscape requires interdisciplinary and transdisciplinary stakeholder interaction processes also known from comparable innovations^21,22^. The model results presented here provide new insights into the relevance of this approach. For the new type of photovoltaics, implementation, and management aspects differ in detail from established photovoltaic applications^3^.

The methodology presented in this study integrates geospatial data from German national authorities with GIS tools to develop a workflow that identifies and ranks field margins according to their suitability for hosting photovoltaic plants in Germany. Methodologically, this study relies on a vector-based GIS workflow that extracts and segments field margins from official landscape datasets. It applies rule-based spatial threshold and suitability criteria (including simplified shading classes), and derives photovoltaic potential based on solar exposure and the assumed module area per meter. The use of corresponding GIS‑based methods to identify optimal agricultural areas for photovoltaic installations is well established in the scientific literature^23^, and similar approaches have been applied to linear structures in the landscape such as highways and railways. For example,^24^ present a Python-based, GIS-driven method for identifying photovoltaic installation sites along highways. This method integrates GIS layers for tunnels, bridges, overpasses, guardrails, and barriers with irradiance and tree-cover density data. It can be used to identify photovoltaic capacity and rank installations based on area, expected yield, distance to the nearest delivery point, and shading. Focusing on Europe’s major roads and railways,^25^ present a further GIS-based framework for identifying photovoltaic potential along transport routes by processing available road and railway datasets and making use of the Photovoltaic Geographical Information System (PVGIS) of the European Commission’s Joint Research Centre (JRC) and a Digital Elevation Model (DEM). Unlike the present study, additional spatial irradiance data is obtained from external data sources rather than being derived from digital landscape data (like ATKIS in this case).

Owing to the nationally harmonised geospatial data structure provided by German authorities and the use of geometry‑based shading calculations, the presented approach can be applied to other regions in Germany without requiring methodological adaptations. The robustness and scientific validity of the workflow were confirmed through ground‑truthing in the Weser‑Ems region using high‑resolution orthophotos (DOPs). Thirty representative field‑margin samples, selected via GIS‑based k‑means clustering, showed 97% agreement with model attributes. Shading analysis further supports the reliability and applicability of this approach to field-margin photovoltaic planning. In principle, applying the model to a different geospatial data basis without ATKIS is feasible, although adjustments to the geospatial and attribute data components may be necessary. The accuracy of the results is critically dependent on the completeness and precision of field‑margin datasets, which, in the absence of such data, could potentially be derived from remote‑sensing imagery using boundary‑detection algorithms (e.g., edge detection), albeit with a slightly higher degree of uncertainty.

The databases used for estimating field margin length and orientation consist of topographical geodata provided by official authorities. These datasets are regularly updated, freely accessible for scientific and non-commercial purposes, and ensure a high level of accuracy and topicality. In particular, the ATKIS dataset provides a reliable and nationally standardised basis for modelling spatial capacities and configurations, ensuring consistency and interoperability across regions. All datasets are subject to the guidelines of the Infrastructure for Spatial Information in Europe (INSPIRE), which stipulate the use of interoperable geodata and a unified spatial reference system (ETRS89) that enables cross-border applications. The reliability of the model, its specification, and data processing was further confirmed through ground-truthing using high-resolution digital orthophotos (DOPs) (cf. Fig. 10).

While the technical robustness and reproducibility of the workflow are evident, the practical realisation of field-margin photovoltaics is influenced by a range of infrastructural and socio-economic factors that extend beyond the scope of geospatial modelling. In particular, technical issues related to grid connection play a role. While the vicinity of buildings grid access is usually easier, the grid connection for smaller, remote field margin photovoltaics is likely to be more difficult compared to solar farm installations that cover larger areas. Furthermore, frequently underestimated cultural and traditional viewpoints of potential adopters require attention. The integrated use of land for renewable energy combined with crop cultivation is a challenge. Farmers will be required to gain expertise in the domain of energy systems, including familiarity with some engineering backgrounds and a certain understanding of the involved (digital) cybernetic implications. The resulting transformation of the farming system may also induce a further trend towards a practical involvement in applied rural technology development and modification of suggested solutions to meet specific local conditions. This trend was already apparent in the ongoing experiments with agrophotovoltaics, systems to gain electric energy with installations above cultivated crops^26^.

The European Union’s administrative regulations and subsidy policies have a strong influence on farmers’ decisions. In the longer term, field margin photovoltaics as a new element would have to find a place in the regulatory framework. So far, wildflower strips combined with additional uses on the same site are not (yet) a part of measures qualifying for subsidies within the Common Agricultural Policy (CAP)^27^.

The full range of technical, economic, and cultural dimensions indicates the necessity for a ground-breaking transformation process in agriculture to cope with the challenges of achieving climate resilience and sustainability. The integration of renewable energies in farming systems can play a stabilising role for the rural sector without competing with food production. While the integration of new technology components involves open questions, the beneficial effects for rural biodiversity can be estimated based on long-established and comparatively well-understood cause-effect lines. Hedgerows, flower strips and, in some strongly wind-exposed landscapes, protective walls around fields are landscape elements with a centuries-old tradition. They are known to play a significant role as habitat connecting structures facilitating the spread of wildlife, and for species that are important for natural pest control (e.g.^28^). In various regions, hedgerows have a special protected status to prevent their removal in the course of land consolidation for the standardised management of larger areas^29^. So far, they contribute only indirectly to agricultural income generation through their stabilising function.

**Table 1:**
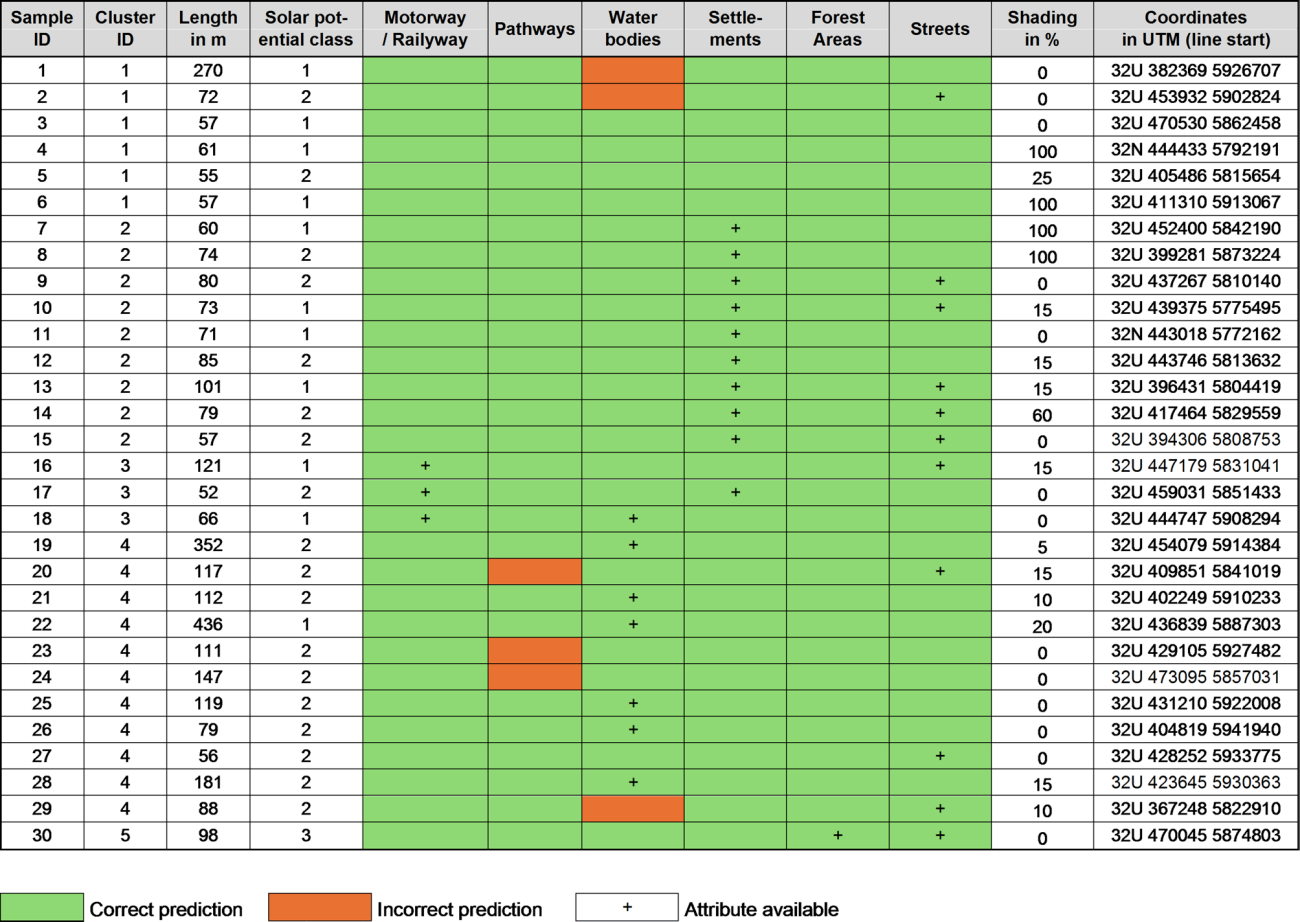
Table of samples and ground truthing results.

Shown are the correct (green) and incorrect (red) predictions of the model’s attributes for the field margins (‘+’ means the attribute is available), checked against DOPs.

It is commonplace, that the economic prospects for farmers, especially the smaller ones, are difficult. This emphasises transformation considerations. Through our model results, we were able to substantiate, that the overall potential of field margin photovoltaics on a regional scale can make a relevant quantitative contribution.

The databases for field margin length and orientation estimations consist of topographical geospatial data from official authorities. They are regularly updated and accessible for scientific purposes. ATKIS data provide a reliable basis for modelling analyses, ensuring consistency and interoperability across regions. The data ensure a high level of accuracy and topicality. They are subject to the guidelines of the Infrastructure for Spatial Information in Europe (INSPIRE), which requires the interoperability of geospatial data and a standardised spatial reference system (ETRS89) that enables cross-border use. The use of DOPs for ground truthing (cf. Fig. 10) confirmed the reliability of the model, its specification, and data processing.

One of the next steps is to use the model results to inform a regional living lab process, i.e. a practical stakeholder discourse to broaden public perception and interaction as proposed in transformation research^30,31^. In the region of Lower Saxony, plans are currently underway to extend conservation measures: Re-wetting of former peatlands currently used for agriculture to stop the release of CO_2_ from drained organic soils. This would require the introduction of alternative forms of cultivation^32^. Photovoltaics could play a central role as a source of income to compensate farmers for the economic loss of abandoning established land use. Land use alternatives and climate change adaptation of the rural economy are becoming key points for the future sustainability of rural communities. We expect that integration of renewable energy systems in locally adapted configurations is a necessary option for the required social-ecological viability perspective.

## Supplementary Information

Below is the link to the electronic supplementary material.

Supplementary Material 1

## Data Availability

The Open Geodata Portal of the State Office for Geoinformation and Land Surveying Lower Saxony (Landesamt für Geoinformation und Landesvermessung Niedersachsen – LGLN) provides free access to all data used in this study: https://ni-lgln-opengeodata.hub.arcgis.com/. The *Supplementary Data S1* includes the relevant ArcGIS Toolbox as well as a standalone Python script version of the workflow, available at https://doi.org/10.5281/zenodo.17498527.
