# Supplementary figures and images for: GIS-based analysis of field margin photovoltaic potential at the landscape level in northwestern Germany
Source: Sci Rep. 2026 Apr 24;16:13394. doi: 10.1038/s41598-026-48425-2 (PMC13109408; doi:10.1038/s41598-026-48425-2)

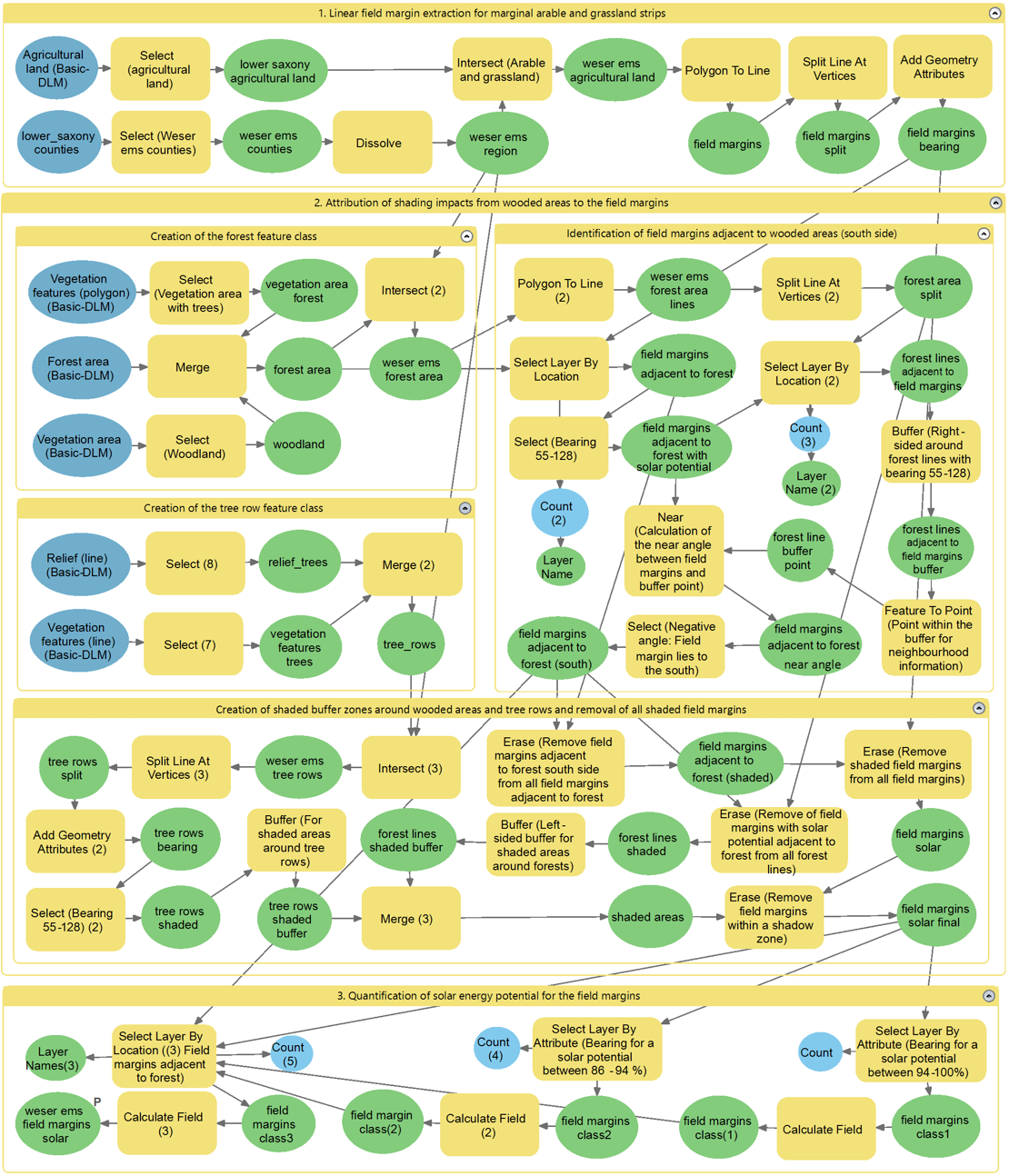

Supplement: Supplementary file 1 — Supplementary Material 1 [file 41598_2026_48425_MOESM1_ESM.zip › Supplementary_Data_S1_Field_Margin_Photovoltaics_Workflow_Foth_2025/modelbuilder_workflow.PNG]
